# Reduced Freezing in Posttraumatic Stress Disorder Patients while Watching Affective Pictures

**DOI:** 10.3389/fpsyt.2017.00039

**Published:** 2017-03-14

**Authors:** Iro Fragkaki, Karin Roelofs, John Stins, Ruud A. Jongedijk, Muriel A. Hagenaars

**Affiliations:** ^1^Behavioural Science Institute, Radboud University, Nijmegen, Netherlands; ^2^Behavioural Science Institute and Donders Institute for Brain Cognition and Behavior, Radboud University, Nijmegen, Netherlands; ^3^Faculty of Behaviour and Movement Sciences, Department of Human Movement Sciences, MOVE Research Institute Amsterdam, VU University, Amsterdam, Netherlands; ^4^Foundation Centrum ′45, Arq Psychotrauma Expert Group, Diemen, Netherlands; ^5^Department of Clinical Psychology, Utrecht University, Utrecht, Netherlands

**Keywords:** freezing, bradycardia, heart rate, posttraumatic stress disorder, body sway

## Abstract

Besides fight and flight responses, animals and humans may respond to threat with freezing, a response characterized by bradycardia and physical immobility. Risk assessment is proposed to be enhanced during freezing to promote optimal decision making. Indeed, healthy participants showed freezing-like responses to threat cues. Posttraumatic stress disorder (PTSD) patients are characterized by hypervigilance and increased threat responsiveness. We propose that threat responses will be characterized by decreased freezing in PTSD, eliminating possibilities for rejecting cognitive distortions, such as harm expectancy, and thereby contributing to the maintenance of the disorder. However, freezing responses have hardly been investigated in PTSD. Using a stabilometric platform to assess body sway as an indicator of freezing-like behavior, we examined whether veterans with PTSD would show diminished freezing responses to unpleasant versus neutral and pleasant pictures. Fourteen PTSD patients and 14 healthy matched controls watched the pictures, while body sway and heart rate (HR) were continuously assessed. Replicating previous findings, healthy controls showed decreased body sway and HR in response to unpleasant pictures, indicative of freezing-like behavior. In contrast, this response pattern was not observed in PTSD patients. The results may indicate a reduced freezing response in PTSD. As reduced freezing may hinder appropriate risk assessment, it may be an important factor in the maintenance of PTSD. Future research might clarify whether impaired freezing is a PTSD-specific or a transdiagnostic symptom, being present in threat-related disorders.

## Introduction

When facing threat, animals as well as humans typically respond with freezing, flight, or fight. A defense cascade of these responses has been described, demonstrating that the selection of the appropriate response is complex and depends on many factors such as threat imminence and escape options (1–5). Although freezing is a routine outcome measure in animal research, research in human stress responses has mostly focused on fight/flight behavior and research on human freezing is still in its infancy. Freezing is a defensive response characterized by reduced physical movement and reduced heart rate (HR) (bradycardia). It is generally considered adaptive as it avoids detection by a predator and enhances action preparation. During freezing, perceptual and attentional processes are optimized. This modulation of information processing could serve to enhance risk assessment and decision making (6). Risk assessment usually occurs after threat detection to assess the stimulus and select the appropriate defense response. It was indeed found to be more frequent during freezing (7). The organism assesses the threat and the environment in order to eventually select the optimal response and prepare for action (8–10).

Recent defense models describe similar defense responses, while adding orienting (at the beginning of the cascade) and tonic immobility (at the very end of the cascade) (8, 10, 11). These models propose distinct functions for each defense response as well as distinct physiological and neurobiological features (10, 11). Freezing is proposed to be different from the other two threat-related immobility responses (orienting and tonic immobility) in several ways. Orienting involves a strong attentional component toward the stimulus, but it is mostly triggered by novelty or moderately intense stimuli, it habituates more rapidly and it is accompanied by small postural adjustments to direct attention toward the stimulus (12). In contrast, freezing does not habituate and can be prolonged, depending on circumstances (8, 13). Tonic immobility occurs later in the defense cascade (circa-strike physical contact), when fight, flight, and freezing are no longer good survival options. It may help survival as well by “playing dead” so that the predator loses interest (8, 10, 14, 15). Although tonic immobility is also characterized by physical immobility, not all physical markers are the same as for freezing. For example, HR increases have been reported for tonic immobility, as well as closed eyes, a relative unresponsiveness, and dissociative symptoms (16–18). Note that dissociation is not considered to be a feature of freezing. It may be a part of the TI response (19, 20), but its mechanism is proposed to be different from TI [cognitive processing would be impeded during dissociation while intact or enhanced during TI (21, 22)]. In humans, self-reported tonic immobility during trauma has been associated with the development of posttraumatic stress disorder (PTSD) (23, 24). The aim of the current study was to investigate freezing behavior in PTSD, so we used a passive viewing paradigm that typically evokes long-lasting freezing behavior (10, 14, 25–27).

Like animals, humans may show freezing too when facing threat. It can be elicited in a laboratory setting by presenting analog threat such as unpleasant pictures, angry faces, and unpleasant films in adults (14, 25–31). Using such a “passive viewing paradigm,” freezing is typically defined by its physical markers: reductions in body sway (assessed by a stabilometric platform) and bradycardia in response to analog threat relative to neutral or pleasant stimuli. Only a few studies have examined individual differences in freezing using the passive viewing paradigm and physical markers for freezing. Freezing was associated with state anxiety (27), insecure attachment (32), and a history of traumatic experiences, with enhanced freezing in those with one trauma and generalized freezing in those with multiple traumas (26). Note that all these findings are in healthy participants though, possibly pointing out the function of freezing as an adequate threat response. That is, enhanced attentional and perceptual processing is adequate when facing visual threat (i.e., affective stimuli) and helps to prepare for appropriate action (9). At this point, our understanding of the role of freezing in psychopathology is very limited.

Posttraumatic stress disorder is characterized by hypervigilance, hyperarousal, and increased responsivity to environmental stimuli (33–35). PTSD patients tend to respond excessively to ambiguous and (non-threatening) trauma reminders, suggesting impaired risk assessment or freezing. That is, PTSD patients perceive all stimuli as threatening and act accordingly with immediate behavioral and physiological action, such as increased sympathetic activity, increased startle response, and increased avoidance (33, 35). This pattern might contribute to the maintenance of PTSD because it inhibits the adaptive risk assessment of stimuli and may lead to persistent maladaptive defense responses. In addition, as a consequence harm expectancy and catastrophic cognitions are not disconfirmed, again contributing to PTSD maintenance. Therefore, automatic defense responses merit further exploration. Moreover, research on automatic defense responses may be highly relevant as it is consistent with animal models (36).

Despite this theoretical relevance, there is limited research on automatic freezing responses in PTSD. There are just a few studies that measured objective immobility responses (i.e., body sway and/or HR) in PTSD patients. Adenauer et al. (37) examined the risk assessment/orienting phase in PTSD while watching affective pictures. HR responses were recorded in war and torture exposed individuals with and without PTSD and healthy controls using a passive viewing paradigm with neutral, pleasant, and unpleasant pictures. Trauma-exposed individuals without PTSD and healthy controls exhibited an initial HR deceleration (in the first 2 s of picture presentation) when watching unpleasant pictures. In contrast, PTSD patients showed almost immediate HR acceleration in response to unpleasant pictures. Moreover, the trauma-exposed individuals without PTSD showed a stronger initial deceleration in response to all pictures. The authors suggested that the orienting response to aversive stimuli was almost absent in PTSD patients, indicating that the defense cascade in PTSD was shifted toward a rapid fight–flight response without any prior exploration of the stimulus. This enhanced fight/flight response might indicate reduced freezing as well. Also, although body sway was not assessed, the findings from Adenauer et al. (37) are in line with those from Hagenaars et al. (26), who found enhanced freezing in response to unpleasant pictures for *healthy* participants who had encountered multiple traumas but had not developed PTSD. Thus, enhanced freezing might indicate resilience.

A study by Volchan et al. (38) examined body sway and HR in PTSD patients and healthy controls in response to autobiographical trauma scripts. They found that subjective reports of tonic immobility during the script were related to reduced body sway and increased HR during 60 s after the script for all the participants irrespective of group. There were no differences between PTSD patients and controls in objective body sway after the script and group differences on HR were not reported. One other study found increased body sway during a stressor (eye closure) in PTSD relative to healthy controls (39). Moreover, self-reported tonic immobility was related to body sway reductions in response to the stressor in both groups. However, note that Volchan et al. (38) measured body sway *after* the trauma script, thus, in the recovery phase and not during threat, and Fragkaki et al. (39) measured body sway during an acute stressor instead of watching threat, and both studies possibly indicated tonic immobility and not freezing.

A few studies have also examined freezing in other psychiatric disorders. One study examined freezing (body sway responses without HR) in patients with panic disorder (PD) and healthy controls with a passive viewing paradigm of neutral, unpleasant, and anxiogenic pictures (40). They found reduced body sway in PD patients compared to controls throughout the experiment (thus independent of picture valence). This decreased body sway was associated with increased anticipatory anxiety as a PD symptom [i.e., the anticipatory anxiety subscale of the Panic-Associated Symptoms Scale (41)]. However, there was no specific effect on unpleasant and anxiogenic pictures (which represent the threatening cues), and HR was not recorded. Freezing responses as indicated by HR deceleration have also been examined in patients with borderline personality disorder (42), yielding an absence of freezing-like responses in patients but not healthy controls while watching unpleasant pictures. Importantly, decreased bradycardia was related to a lower tolerance of distress, i.e., a higher tendency to avoid or suppress negative emotions, which may confirm the process of taxing the threat during freezing. The next step is to examine freezing behavior in PTSD patients using both objective markers (body sway and HR) and a clear “freezing” paradigm (passive viewing).

Finally, postural changes are sensitive and show great fluctuations over time. As a consequence, results are usually reported as a mean over a longer period of time (1 minute), or as an event-related response. Freezing-related changes in HR are more stable and sustained (10, 43). This pattern was confirmed in a study by Hagenaars et al. (14), who examined specific time response patterns throughout the 60 s of neutral, pleasant, and unpleasant films. Indeed, they found an initial deceleration in HR during unpleasant films compared to neutral and pleasant films from 0 to 30 s that was sustained until the end of the film. With respect to body sway, there was an overall effect of emotion indicating that body sway decreased during unpleasant films but there was no main effect of time. This finding highlights a sustained and relatively slow decrease in HR in response to aversive stimuli in healthy individuals.

In summary, although theoretically there should be a pattern of reduced freezing in PTSD, empirical evidence is limited. The present study, therefore, aimed to examine freezing-like patterns (indicated by reduced body sway and HR) in veterans with PTSD, relative to a healthy control group in response to affective picture viewing focusing on overall effects and specific time patterns. Controls were expected to show freezing-like behavior, i.e., decreased body sway and HR in response to unpleasant (versus neutral and pleasant) pictures. More specifically, we expected a HR deceleration in the first 30 s in healthy controls. We also anticipated an overall effect of picture valence on body sway in healthy controls, namely a decrease in body sway during unpleasant pictures. Patients with PTSD were expected to show reduced freezing, indicated by attenuated decreases in HR and a lack of body sway reduction during unpleasant picture viewing.

## Materials and Methods

### Participants

The study included 19 veterans with a principle diagnosis of PTSD and 15 healthy controls. Patients with PTSD were recruited from a Dutch national mental health care organization specialized in PTSD. Controls were recruited via advertisements. The analyses included participants with complete and valid data for all time point measurements of body sway and HR, leaving a total sample of 14 PTSD patients and 14 healthy controls. Comorbidity was high: depressive disorder (*n* = 10), substance abuse disorder (*n* = 6), PD with agoraphobia (*n* = 2), impulse control disorder (*n* = 1), somatization disorder (*n* = 1), conversion disorder (*n* = 1), and personality disorder NAO (*n* = 2), and schizotypical personality disorder (*n* = 1). Three PTSD patients had no comorbid disorder. Nine PTSD patients received medication at the time of the study: antidepressants (*n* = 6), sedatives (mainly benzodiazepines; *n* = 8), and antipsychotics (*n* = 4). Controls were healthy and did not use medication. All participants were male and matched on age (see Table 1 for descriptives). Participants received a financial reimbursement (15 euro) for their participation. Vision was normal or corrected-to-normal in all participants. The study was conducted in accordance with the Declaration of Helsinki and was approved by the Ethics Committee of Leiden University. All participants gave written informed consent.

**Table 1:** **Descriptive characteristics of posttraumatic stress disorder (PTSD) patients and controls**.

	PTSD		Controls	
	M	SD	M	SD
Age	41.640	8.563	44.640	10.775
STAI-S***	44.071	9.980	30.000	4.961
STAI-T***	51.769	13.534	30.285	5.862
BDI-II***	26.000	14.523	5.140	4.975

	***n* (*x*%)**		***n* (*x*%)**	

Educational level*				
Low	5 (45.5)		2 (14.3)	
Middle	4 (36.4)		2 (14.3)	
High	2 (18.2)		10 (71.4)	

*STAI-S, Spielberger State Anxiety Inventory; STAI-T, Spielberger Trait Anxiety Inventory; BDI-II, Beck Depression Inventory-II*.

*p < 0.05.

***p < 0.001.

### Apparatus and Material

#### Heart Rate

Heart rate was measured with a polar band (HR Telemetry Systems) attached at the height of the sternum. The signal was converted to beats per minute with LabVIEW (National Instruments Corp., Austin, TX, USA). We measured HR continuously during picture viewing. Mean HR was calculated for each stimulus block (neutral, pleasant, and unpleasant). Moreover, 3 s intervals were calculated for each picture block, consistent with previous research (10, 14), in order to examine (group differences in) time course.

#### Body Sway

Body sway was assessed using a 1m × 1m stabilometric platform at a sample frequency of 100 Hz. Center of pressure (COP) excursions in anterior–posterior (AP) and medio-lateral (ML) directions were recorded. Standard deviations of the time series were calculated separately for the AP and ML components of the COP. We used the standard deviations in the AP direction in our analyses because AP direction has been found to be most sensitive to trauma-related approach–avoidance responses and the position as required in this experiment (feet 30 cm apart) is more susceptible to movements in the AP direction (26, 27). In addition, we intended to keep the number of tests to a minimum and the examination of the AP direction is commonly used as an outcome measure in prosturography (25–27). Lower SD-AP scores indicated increased immobility.

#### Pictorial Stimuli

Three sets of stimuli were selected from the International Affective Picture System [IAPS (44)].^1^ Each set comprised 20 pictures of neutral, pleasant, or unpleasant pictures. Similar pictures were used in previous studies (25, 26, 37, 40, 45) in order to facilitate comparisons across studies. Stimuli were presented full screen at eye-height on a 17″ height adjustable computer screen, approximately 1 m in front of the participant with a visual angle (height × width in degrees) of 15.5° × 10.8°.

#### Picture Ratings

Participants rated the pictures on pleasantness and arousal on the 9-point Likert scale of the IAPS rating system, with higher scores indicating that the picture was perceived as more unpleasant (*1* = *pleasant, 9* = *unpleasant*) and less arousing (*1* = *high arousal, 9* = *low arousal*).

#### PTSD Diagnostic Status

Posttraumatic stress disorder symptoms were assessed with the Clinician Administered PTSD Scale [CAPS-IV (46)]. The CAPS-IV is a structured clinical interview that assesses the presence of DSM-IV criteria for PTSD. Frequency and intensity of PTSD symptoms are scored in a 5-point Likert scale (0–4) that can be summed to a 9-point scale (0–8) score for each symptom. The CAPS-IV has well-established psychometric properties with excellent interrater diagnostic agreement and internal consistency (α = 0.94), good test-retest reliability for the three symptom clusters (*r* = 0.77 to 0.96), and adequate concurrent validity with other measures of PTSD (47, 48). The CAPS was administered by a registered psychotherapist who was trained in the CAPS.

#### State and Trait Anxiety

Anxiety was assessed with the state and trait anxiety scale of the Spielberger State-Trait Anxiety Inventory [STAI (49)]. STAI has two subscales, state and trait anxiety, and each contains 20 self-report items measuring how anxious the participants feel at the present moment (state) in a 4-point Likert scale from 1 (*not at all*) to 4 (*very much so*) or how anxious they generally feel (trait) from 1 (*almost never*) to 4 (*almost always*). The total score is the sum of the items and ranges from 20 to 80 with higher score indicating higher level of state or trait anxiety. STAI is a widely used measurement of anxiety with high internal consistency (α = 0.90), good test–retest reliability (*r* = 0.70–0.76), and concurrent validity with other measures of anxiety (49).

#### Depressive Symptoms

The Beck Depression Inventory-II [BDI (50, 51)] was administered to assess depressive symptoms. The BDI-II is a 21-item self-report measurement of depressive symptoms according to the Diagnostic and Statistical Manual of Mental Disorders, Fourth Edition [DSM-IV (52)] criteria. Each item includes four statements that describe an emotional state or behavior displayed in increasing intensity, ranging from absence of symptoms (0) to severe symptoms (3). It is a widely used instrument for depressive symptoms with established psychometric properties in clinical and community samples (53, 54).

### Procedure

Prior to the beginning of the experiment, participants were assessed for PTSD and anxiety and depressive symptoms and then the polar band was attached to their sternum. The experiment took place in a dimly lit room and the participants were asked to step onto the stabilometric platform and pay attention to the instructions displayed on the monitor in front of them. The participants were instructed to remove their shoes, stand upright with their feet 30 cm apart on the middle of the stabilometric platform, and stand still with their hands hanging relaxed alongside their body. They were asked to watch the sequence of images displayed on the monitor while maintaining this position. Before the experimental task, there was a brief practice trial with the presentation of letters.

The experimental task comprised passive viewing of the 60 emotional pictures described above. Pictures were displayed in three blocks of 20 pictures of each valence. The order of the blocks was counterbalanced and the order of the pictures within each block was randomized. Each picture was displayed for 3 s without between picture interval, resulting in a total duration of 60 s per block, consistent with previous studies (25, 26, 29). A 5 s black screen followed by a 2 s white fixation cross was presented before each block. The total time viewing was 4 min 21 s (60 s practice trial + 201 s picture viewing). Subsequently, participants completed picture ratings. The CAPS-IV was administered at the end of the experiment to avoid negative mood induction.

### Statistical Analyses

Analyses of postugraphic data were conducted with MATLAB (MathWorks, Natick, MA, USA) and further statistical analyses were performed with the Statistical Package for Social Sciences (IBM SPSS 20.0). The time series were filtered with a second-order low-pass Butterworth filter with a cutoff frequency of 10 Hz. Baseline differences were examined with independent *t*-tests or Mann–Whitney tests in case of non-normal distributions, and adjusted df were reported when the assumption of equal variances was not met. Freezing was defined as a decrease in body sway and HR from neutral to unpleasant (SD-AP and HR during neutral pictures minus SD-AP and HR during unpleasant pictures) and from pleasant to unpleasant (SD-AP and HR during pleasant pictures minus SD-AP and HR during unpleasant pictures). We conducted a repeated measures analysis of variance (rmANOVA) with Group (Control, PTSD) as the between-subjects factor, Valence (neutral, pleasant, and unpleasant) and Time (20 × 3 s) as within subjects factor, and SD-AP and HR as dependent variables. All tests were two tailed with the significance level set at 0.05.

## Results

### Baseline Differences

Table 1 presents the age, educational level, and anxiety scores for the two groups. Groups did not differ in age [*t*(26) = −0.816, *p* = 0.422], but healthy controls were higher educated than PTSD patients [χ^2^ (2, *N* = 25) = 7.027, *p* = 0.030]. PTSD patients scored higher on STAI-state [*t*(19) = 4.724, *p* < 0.001], STAI-trait (*U* = 20.00, *p* < 0.001), BDI-II (*U* = 21.00, *p* < 0.001) and had a mean CAPS-IV score of 87.14 (SD = 13.95).

### Posturography

**Figure 1:**
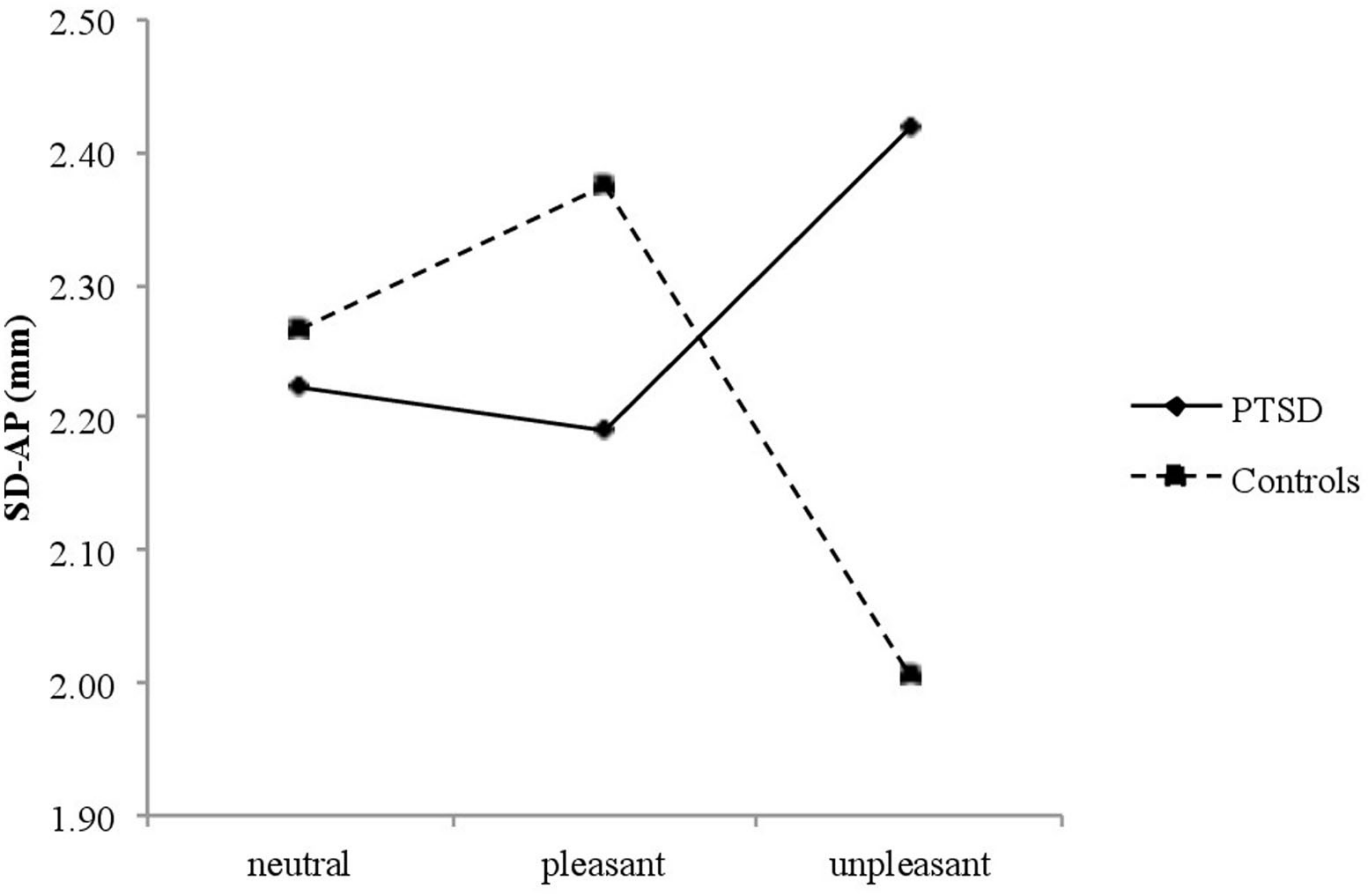
**Mean SD-AP for neutral, pleasant, and unpleasant pictures in posttraumatic stress disorder (PTSD) patients and controls**.

The rmANOVA revealed a significant Group × Valence interaction on SD-AP [*F*(2, 52) = 3.587, *p* = 0.035, $\eta_{\text{p}}^{2}=0.121$], suggesting that affective pictures elicited different body sway responses in PTSD patients and controls. As there were no effects of time, *post hoc* rmANOVA were conducted for each group with Valence (mean SD-AP for each picture block: neutral, pleasant, and unpleasant) as dependent variable, in order to examine specific patterns of body sway within each group (25–27). There was a significant Valence effect for healthy controls [*F*(2, 26) = 5.308, *p* = 0.012, $\eta_{\text{p}}^{2}=0.290$], who showed decreased SD-AP for unpleasant versus neutral (*p* = 0.023) and pleasant (*p* = 0.011) pictures, but not for neutral versus pleasant pictures (*p* = 0.470). In contrast, PTSD patients did not exhibit significant differences in SD-AP [*F*(2, 26) = 0.756, *p* = 0.480] (see Figure 1).

The main effects of Group [*F*(1, 26) = 0.094, *p* = 0.762], Valence [*F*(2, 52) = 0.214, *p* = 0.808], and Time [*F*(19, 494) = 0.1.536, *p* = 0.069] were not significant. There were neither significant interactions for Time × Group, Valence × Time nor a significant Valence × Time × Group interaction (all *F*s < 1.265, all *p*s > 0.271).

### Heart Rate

**Figure 2:**
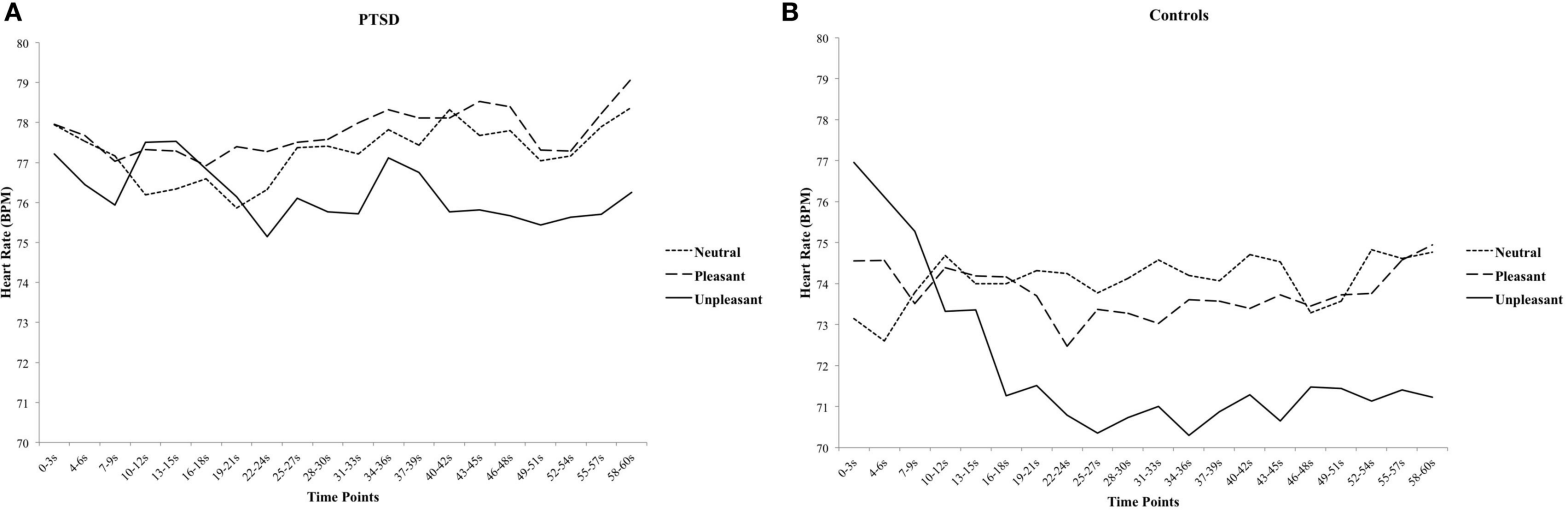
**The course of heart rate for neutral, pleasant, and unpleasant pictures in posttraumatic stress disorder (PTSD) patients (A) and healthy controls (B) in 3-s blocks**.

For HR, there was a significant Group × Valence × Time interaction, [*F*(38, 988) = 1.520, *p* = 0.024, $\eta_{\text{p}}^{2}=0.055$], suggesting that affective pictures elicited distinct HR courses between groups. Separate rmANOVA for each group were conducted to examine the specific patterns in HR for PTSD and healthy controls. There was a significant Valence × Time interaction in healthy controls [*F*(38, 494) = 2.870, *p* < 0.001, $\eta_{\text{p}}^{2}=0.181$], showing an initial deceleration in HR during unpleasant pictures, and the reduced HR was sustained until the end of the block (Figure 2). In contrast, there was no significant Valence × Time interaction in PTSD patients, [*F*(38, 494) = 0.798, *p* = 0.802] indicating an attenuated freezing-like response to unpleasant pictures (Figure 2).

There was a main effect of Valence [*F*(2, 52) = 4.103, *p* = 0.022, $\eta_{\text{p}}^{2}=0.136$] and Time [*F*(19, 494) = 2.259, *p* = 0.002, $\eta_{\text{p}}^{2}=0.080$], indicating that there was an overall decrease in HR from neutral and pleasant to unpleasant pictures and over time. There was no main effect of Group [*F*(1, 26) = 0.738, *p* = 0.398]. None of the other two-way interactions were significant (all *F*s < 1.403, all *p*s > 0.119).

### Subjective Picture Ratings

A 2 (Group) × 3 (Picture Valence) rmANOVA showed a significant main effect of Picture Valence on pleasantness ratings [*F*(2, 52) = 125.122, *p* < 0.001, $\eta_{\text{p}}^{2}=0.828$]. *Post hoc* comparisons showed significant differences between all emotion categories with pleasant pictures perceived as most pleasant and unpleasant pictures as most unpleasant (neutral: M = 5.153, SD = 0.940, pleasant: M = 3.791, SD = 0.994, unpleasant: M = 7.619, SD = 0.949; all *p*s < 0.001). There was no Group × Picture Valence interaction [*F*(2, 52) = 1.095, *p* = 0.342] indicating that pleasantness ratings did not differ between PTSD patients and Controls. There was also a main effect of Picture Valence on arousal ratings [*F*(2, 52) = 76.956, *p* < 0.001, $\eta_{\text{p}}^{2}=0.747$]. *Post hoc* comparisons showed significant differences between all emotion categories with neutral pictures eliciting the lowest arousal and unpleasant pictures the highest arousal levels (neutral: M = 7.955, SD = 1.309, pleasant: M = 6.376, SD = 1.631, unpleasant: M = 4.014, SD = 2.170; all *p*s < 0.001). There was no Group × Picture Valence interaction effect [*F*(2, 52) = 1.256, *p* = 0.293] indicating that picture arousal ratings did not differ between PTSD patients and Controls.

## Discussion

The aim of this study was to investigate whether PTSD is associated with reduced freezing in response to affective picture viewing. We hypothesized that healthy controls but not PTSD patients would exhibit freezing in response to unpleasant pictures, indicated by reduced body sway and HR deceleration during the first 30 s. The results confirmed our hypothesis as healthy participants showed freezing-like behavior (reduced body sway and HR) in response to unpleasant pictures. Particularly, healthy controls exhibited an initial deceleration in HR during the first 30 s of the unpleasant pictures block and a sustained reduced HR until the end of the block. Body sway showed an overall decrease in healthy controls during the unpleasant pictures block without specific time patterns. This freezing-like behavior was not found in PTSD patients, who did not show differences in body sway or HR in response to unpleasant pictures relative to neutral and pleasant pictures.

These findings are in line with previous studies demonstrating a freezing pattern in response to aversive stimuli in healthy individuals (10, 14, 25–27, 29–31). Our findings also match with two studies that found increased body sway in PTSD patients compared to controls in response to eye closure and no bradycardia in patients with borderline personality disorder compared to controls in response to unpleasant pictures (39, 42). Overall, our study demonstrates reduced freezing in PTSD measured by *both* its physical markers (reduced body sway and bradycardia). Together, these findings might indicate elevated fight–flight behavior and attenuated freezing in response to (laboratory) stressors (37). In addition, we replicated the HR pattern found by Hagenaars et al. (14) in controls, with HR deceleration in the first half and a sustained reduced HR in the second half. This finding is of great importance as it highlights freezing as a sustained defense response and distinguishes it from orienting.

In contrast, Volchan et al. (38) did not find differences in body sway between PTSD patients and healthy controls after a trauma script. Note though, that they measured body sway *after* the stressor had ended and not *during* the stressor. Recovery or reactivity might take place in this time frame, rather than risk assessment and freezing. The authors indeed do not refer to freezing but to tonic immobility, which is a distinct defense response. Speculatively, our and previous data may indicate that *reduced* freezing but *increased* tonic immobility is associated with psychopathology. Alternatively, tonic immobility may be associated with PTSD *development*, while freezing may be associated with PTSD *maintenance*. Critically, note that methodological differences may also account for seemingly contradictory or counterintuitive findings: freezing is assessed with physical indicators (body sway and/or HR reductions), while tonic immobility is measured retrospectively with self-report measures. Our findings also seem to contradict those of Lopes et al. (40), who showed reduced body sway in PD patients compared to controls in response to picture viewing. However, besides that PD and not PTSD patients were included, this effect was observed throughout the experiment and not particularly in response to unpleasant or anxiogenic pictures.

The absence of freezing in PTSD is in accordance with the theoretical model of PTSD. PTSD patients exhibit hypervigilance, hyperarousal, and increased responsivity to environmental stimuli (33–35). Moreover, over-generalization of threat, dysfunctional extinction learning, and impaired fear inhibitory learning are core characteristics of PTSD (55–59). PTSD patients tend to perceive aversive stimuli or trauma reminders as a real threat and respond with increased stress. We propose that this behavioral pattern might be related to impaired risk assessment. That is, PTSD patients do not assess possible threat cues sufficiently but they immediately experience them as acutely threatening and react accordingly, thereby failing to disconfirm cognitive distortions of harm expectancy (56). This may result in a vicious circle: increased threat perception may lead to impaired risk assessment and attenuated freezing, which in turn, leads to the persistence of cognitive distortions. These distortions in turn fuel threat perception, thus perpetuating this pattern. This vicious circle might play a substantial role in the maintenance of PTSD as well as the effectiveness of treatment. Impaired freezing may be an important part of the vicious circle and thereby a possible impediment for treatment. Speculatively, an effort to restore adaptive freezing responses might be helpful to prevent excessive reactions to ambiguous stimuli, improve risk assessment, and contribute to a more adaptive cognitive processing of external stimuli.

This study was a first exploration of actual, objectively measured freezing responses in PTSD patients. However, limitations include the small sample and the specificity of the PTSD patients, namely male veterans. It is of great importance to further investigate whether impaired freezing is prevalent in distinct PTSD groups (such as female sexual victims, victims of natural disasters, victims of ongoing threat), and whether there are differences between PTSD patients and trauma-exposed individuals without PTSD. In addition, the independent influence of PTSD-relevant factors might be addressed in future studies, such as dissociation, tonic immobility, substance abuse, depression, and alexythimia. For example, distinct emotional processing patterns and stress responses have been found for PTSD and dissociative PTSD (60, 61) and PTSD with high or low peritraumatic TI (39). Most importantly, impaired freezing might be related not only to PTSD but also to other psychiatric threat-related disorders as well, such as anxiety disorders and borderline personality disorder. Reduced bradycardia (as an indicator of freezing) has indeed been reported for patients with borderline personality disorder (42). Speculatively, impaired freezing may be a transdiagnostic symptom, reflecting a disordered downregulation of stress responses or anxiety-related reduced risk taxation and automatic avoidance behavior (62). Finally, PTSD was severe in our sample, as indicated by high CAPS scores, frequent comorbidity (with 64.3% medication intake), and high anxiety levels. Future studies might control for comorbidity and medication, although that would be practically challenging as complex comorbidity and medication intake in PTSD is the rule rather than the exception. For example, lifetime comorbidity in PTSD was found for 88.3% for men and for 79% for women in the National Comorbidity Survey (63) and PTSD patients more often had >3 than 1 comorbid diagnoses (64).

Another intriguing question is whether impaired freezing could be a specific *and* a transdiagnostic symptom. It is possible that freezing is impaired in all threat-related disorders (transdiagnostic) but only in response to disorder relevant stimuli (specific). For instance, impaired freezing could be observed in response to stimuli of spiders in patients with spider phobia or in response to angry faces in patients with social phobia. Therefore, it could be especially useful for future research to include different psychiatric disorders and threat-related stimuli specifically associated with each disorder in order to shed light on the role of freezing in PTSD and threat-related disorders in general. The same is true within PTSD patients: future research should explore whether automatic defense responses are trauma specific (e.g., rape-related pictures for sexual assault victims with PTSD) or non-specific (e.g., war-related pictures for sexual assault victims with PTSD). Finally, HR variability is often used as a stress measure. Although it was found to respond to arousal rather than valence (65) and has never been used as a freezing indicator, it may be interesting to start exploring it as an additional measure.

In conclusion, our study is the first step in examining freezing responses in PTSD patients measured by its two physical markers (body sway and HR). Whereas healthy controls exhibited freezing (reduced body sway and bradycardia) in response to unpleasant pictures, PTSD patients did not exhibit the same pattern. We propose that freezing is impaired in PTSD, indicating that PTSD patients have attenuated risk assessment of the environmental stimuli, which might reinforce the pattern of threat perception, hypervigilance, and hyperarousal and consequently contribute to the maintenance of the disorder. Future research is urged to replicate our study in larger samples and investigate whether impaired freezing is a PTSD-specific, a transdiagnostic symptom or both in threat-related and anxiety disorders in response to specific threat-related stimuli.

## Author Contributions

IF contributed to the analysis and interpretation of the data, drafted and revised the manuscript, agreed to the final version of the manuscript, and agreed to be accountable to all aspects of this work. KR, JS, and RJ contributed to the design of the study, interpreted the data, prepared the raw data (JS), revised the manuscript, agreed to the final version of the manuscript, and agreed to be accountable to all aspects of this work. MH contributed to the conception and design of the study, analyzed and interpreted the data, drafted and revised the manuscript, agreed to the final version of the manuscript, and agreed to be accountable to all aspects of this work.

## Conflict of Interest Statement

The authors declare that the research was conducted in the absence of any commercial or financial relationships that could be construed as a potential conflict of interest.

## Footnotes

^1^IAPS catalog numbers for pictures used in this study are: Neutral: 7000, 7002, 7004, 7006, 7009, 7010, 7020, 7025, 7030, 7031, 7035, 7040, 7050, 7052, 7060, 7080, −7090, 7150, 7175, 7211; Pleasant: 8021, 8032, 8034, 8040, 8041, 8090, 8161, 8186, 8190, 8192, 8200, 8210, 8300, 8370, 8400, 8460, 8465, 8467, 8470, 8620; Unpleasant: 3000, 3010, 3030, 3051, 3053, 3060, 3061, 3062, 3063, 3064, 3069, 3080, 3100, 3102, 3110, 3130 3140, 3150, 3261, 3400.
